# Prevalence and Predictors of Carpal Tunnel Syndrome Symptoms Among Teachers in Jazan: A Cross-Sectional Study

**DOI:** 10.7759/cureus.68458

**Published:** 2024-09-02

**Authors:** Zenat Khired, Ali M Shawish, Mohammed E Mojiri, Ayman M Albarrati, Alhassan H Hobani, Hatem A Madkhali, Ali J Hakami, Khowlah A Adawi, Sultan M Hakami, Layla B Hakami, Khalid Y Muqri, Alyazid Y Awaji, Fatimah E Ageeli, Anas A Sayegh

**Affiliations:** 1 Department of Surgery, Jazan University, Jazan, SAU; 2 College of Medicine, Jazan University, Jazan, SAU; 3 College of Applied Medical Sciences, Jazan University, Jazan, SAU; 4 Department of Orthopedic Surgery, King Fahad Central Hospital, Jazan, SAU; 5 Department of Plastic and Reconstructive Surgery, King Fahad Central Hospital, Jazan, SAU; 6 Department of General Surgery, King Fahad Central Hospital, Jazan, SAU

**Keywords:** teachers, carpal tunnel syndrome, electrophysiology, musculoskeletal pain, wrist pain

## Abstract

Background

Carpal tunnel syndrome (CTS) is a common musculoskeletal condition of the hand and wrist frequently associated with repetitive hand motion and environmental considerations. Teachers are more likely to acquire CTS because of their lengthy writing and computer use. This study aimed to determine the prevalence of CTS symptoms and related variables among schoolteachers in Jazan, Saudi Arabia.

Methods

This study was conducted as a cross-sectional survey of teachers in Jazan, utilizing an online platform for data collection. The Boston Carpal Tunnel Syndrome Questionnaire (BCTQ) was the primary tool used to determine symptom intensity and functional status. The data were rigorously analyzed using a range of statistical methods, including descriptive statistics, the Mann-Whitney U test, the Kruskal-Wallis test, Spearman's correlation, the chi-square test, Fisher's exact test, and binary logistic regression, ensuring the robustness of the findings.

Results

The study comprised 336 schoolteachers with a mean age of 43.3 ± 6.5 years, of whom 58.0% were female and 42.0% were male. About 8.0% of instructors reported CTS symptoms. Female gender (median Symptom Severity Scale (SSS): 15.0 vs. 12.0, p < 0.001; median Functional Status Scale (FSS): 8.0 vs. 8.0, p < 0.001), increased time spent writing (r = 0.237, p < 0.001 for SSS; r = 0.217, p < 0.001 for FSS), and presence of comorbidities such as diabetes (median SSS: 16.0, p = 0.002; median FSS: 8.0, p = 0.001) had a negative correlation with symptom severity (r = -0.174, p = 0.002) and functional impairment (r = -0.141, p = 0.011). Surgical therapy (median SSS: 32.0; median FSS: 24.0; p<0.001) and post-treatment symptom recurrence (median SSS: 28.0; median FSS: 22.0; p<0.001) were associated with increased severity and disability. According to binary logistic regression, increased writing time significantly predicted CTS diagnosis (OR = 1.151, 95% CI: 1.024-1.295, p = 0.018).

Conclusion

CTS symptoms are common among Jazan teachers, and various sociodemographic, vocational, and clinical variables influence their intensity and functional status. Ergonomic treatment, early identification, and suitable management measures are critical for preventing and mitigating the effects of CTS among teachers. Additional research is required to develop focused therapies and enhance the results of this occupational group.

## Introduction

There are numerous musculoskeletal disorders (MSDs) among schoolteachers. They include many inflammatory and degenerative conditions affecting the muscles, peripheral nerves (carpal tunnel syndrome (CTS), tendons (tenosynovitis), bones, joints, and ligaments. This condition may be caused or aggravated by work tasks [1]. Prolonged desk work, extended standing in class, repetitive overhead writing on the board, prolonged sitting from frequent reading, lesson planning, and Internet use were the significant risk factors for MSD among teachers [2]. CTS is a common musculoskeletal disorder. CTS occurs when the median nerve is squeezed or compressed as it travels through the wrists. The clinical manifestations of CTS typically consist of a combination of clinical assessment of signs (e.g., Phalen's sign, Tinel's sign), symptoms (e.g., numbness, tingling, night pain, and paresthesia), and nerve conduction velocity (NCV) testing of the median nerve across the carpal tunnel [3]. However, high prevalence rates of CTS have been reported in certain occupations. An estimated 4% and 5% of people suffer from CTS worldwide, with the most susceptible population being elderly individuals aged between 40 and 60 years [4].

Several individual, ergonomic, and occupational factors, including body mass index (BMI), female gender, repeated wrist flexion and extension, wrist movements for longer than 30 seconds, and more than 50% of the workday spent in repetitive movement patterns, have been linked to the development of CTS in teachers [5]. Repeated flexion and extension of the wrist and other wrist movements associated with long hours spent grading assignments, preparing lessons, performing non-teaching clerical duties, attending developmental courses, and participating in extracurricular activities with students are among the numerous ergonomic and work-related factors that predispose this population to CTS [6]. Furthermore, the rising incidence of metabolic illnesses such as diabetes, hyperthyroidism, and obesity has increased the demand for global healthcare systems, and these conditions are considered risk factors for CTS, particularly in stressful employment settings [7,8]. A thorough history and examination that detects typical symptoms and provoking circumstances enhances the chances of a CTS diagnosis [8]. Nerve conduction tests can be employed in individuals with an intermediate pre-test likelihood or unusual presentation. They can also be used to measure and stratify disease severity, which can help in future treatment decisions [8]. 

The prevalence of CTS among teachers has been reported by Stevens et al. and Younis et al. with considerably disparate estimates of 2.9% and 62%, respectively [9,10]. Given the rise in MSDs among teachers, particularly hand and wrist pain, we reasoned that CTS, a frequent MSD, would be similarly prevalent among instructors [6,10]. Additionally, a study conducted in Riyadh showed that the prevalence of moderate-to-severe CTS symptoms was 40.0%, and self-reported CTS was 9.1% [6]. However, there is a lack of research on the prevalence and factors associated with CTS among teachers in Saudi Arabia, particularly in the Jazan region. This study aimed to address this gap in knowledge by estimating the prevalence of CTS symptoms among schoolteachers in Jazan and identifying the sociodemographic, occupational, and clinical factors associated with symptom severity and functional status.

## Materials and methods

Study design

A cross-sectional study was conducted from April 2024 to July 2024 among teachers in Jazan, a region south of Saudi Arabia with 13 governorates and more than 1,500,000 people distributed in different towns and villages. The study population consisted of male and female teachers working in Jazan for at least one year. This study aimed to estimate the prevalence of CTS symptoms and assess the associated factors among schoolteachers working in this region.

Sample size and sampling method

The sample size for this study was evaluated using the Raosoft sample size calculator [11]. A total of 332 participants were needed to reach a 95% confidence interval and a 5% margin of error. The target sample size was expected to be more than 332. Our study included 336 participants who responded to the survey including male and female teachers. Participants were chosen using convenient sampling among teachers in the Jazan region. The governorates of 6-7 were selected, and then from towns and villages within these governorates, the sample was chosen randomly to ensure the representativeness of the Jazan region teachers.

Inclusion and exclusion criteria

Male and female teachers who had worked for at least a year in Jazan and agreed to participate in the study were included. Teachers with a history of orthopedic trauma or congenital disorders of the wrist, those who did not meet the eligibility criteria (i.e., were not teachers or did not work in Jazan), and those who refused to participate were excluded. These criteria were set to ensure that the study population was representative of the target population and to minimize potential confounding factors.

Data collection

An online survey was distributed through social media applications (X, WhatsApp, and Telegram) to the members of teachers' groups in Jazan. The survey collected demographic information, including age, gender, pregnancy status (for female participants), height, weight, social status, tobacco use, exercise habits, hand dominance, current work status, level of education, subjects taught, and mode of teaching (on-site or virtual). Additionally, the survey gathered data on the years spent teaching and daily time spent writing using a pen, keyboard, and blackboard. The participants were also asked about any clinical diagnosis of CTS by a doctor and other medical comorbidities that could correlate with CTS symptoms.

Instruments

The Boston Carpal Tunnel Syndrome Questionnaire (BCTQ) was used to assess the symptoms and functional status of CTS among teachers. The BCTQ is a validated and reliable tool widely used to determine CTS. It consists of Symptom Severity Scale (SSS) and the Functional Status Scale (FSS). The SSS includes 11 questions evaluating the severity of symptoms such as pain, numbness, and tingling in the hand or wrist, rated on a five-point Likert scale ranging from 1 (average) to 5 (very severe). The total SSS score ranged from 11 to 55, with higher scores indicating more severe symptoms. The FSS comprises eight questions measuring difficulty in performing daily activities, rated on a five-point Likert scale ranging from 1 (no difficulty) to 5 (cannot perform the activity at all due to symptoms). The total FSS score ranged from 8 to 40, with higher scores reflecting more significant functional impairment. The Arabic version of the BCTQ, which was previously validated, was used in this study to ensure cultural appropriateness and understanding among the participants [6].

Data analysis

The analysis used descriptive and inferential statistics. Descriptive statistics were used to summarize and describe the study participants' characteristics and responses. Frequencies and percentages were calculated for categorical variables such as participant responses to the BCTQ. The means, standard deviations, medians, and interquartile ranges were calculated and tabulated for continuous variables.

The BCTQ, a crucial tool in CTS assessment, was used to evaluate symptoms and functional status among teachers. Comprising two parts, the SSS and the FSS, it provides a comprehensive understanding of the condition. The SSS, a vital component of the BCTQ, includes 11 questions that gauge the severity of symptoms. Each item is rated on a practical five-point Likert scale, allowing for a clear assessment of the severity levels, from 1 (normal) to 5 (very serious).

The FSS comprises eight questions that measure the difficulty in performing daily activities, such as writing, buttoning clothes, and gripping a telephone handle. Each item is rated on a five-point Likert scale ranging from 1 (no difficulty) to 5 (cannot perform the activity due to symptoms). The total FSS score was obtained by summing the scores of the eight items, yielding a possible range from 8 to 40. Higher scores reflect more significant functional impairment. The resulting SSS and FSS scores were used in subsequent statistical analyses to determine the study participants' prevalence and predictors of CTS.

For data analysis, we employed a range of statistical tests. The Mann-Whitney U and Kruskal-Wallis tests were conducted to compare the total SSS and FSS scores among the different categorical groups. Spearman's rho correlation was used to determine the correlation of continuous variables with the scores. These non-parametric tests were chosen owing to the normal distribution of the scores assessed by applying the Kolmogorov-Smirnov test (p < 0.05). Moreover, the chi-square test and Fisher exact test (where the expected count in cells was less than 5) were used to find the association of categorical factors with cases already diagnosed with carpel tunnel syndrome. A univariate binary logistic regression model was created for continuous factors, using continuous variables as predictors. The significance level for all statistical tests was set at p < 0.05, indicating a 95% confidence interval. All statistical calculations were performed using IBM SPSS version 27.0.1, ensuring the robustness and reliability of our findings.

## Results

A total of 336 participants were included in this study. The mean age of the participants was 43.3 ± 6.5 years, with an average height of 158.7 ± 18.2 cm and weight of 73.2 ± 15.1 kg. Most participants were female (n = 195, 58.0%), and only four (2.1%) were pregnant among the female participants. Most of the participants were married (n = 284, 84.5%), 34 (10.1%) were single, and 18 (5.4%) were divorced or widowed. Regarding lifestyle factors, 27 (8.0%) participants were smokers, and 169 (50.3%) reported engaging in regular physical activity. Most participants were right-handed (n = 310, 92.3%). Most participants taught in person (n = 328, 97.6%). The distribution of teaching levels was as follows: 145 (43.2%) taught at the primary stage, 113 (33.6%) at high school, 74 (22.0%) at the intermediate stage, and four (1.2%) at kindergarten. The mean duration of employment was 18.1 ± 8.3 years, and participants reported spending an average of 3.2 ± 2.6 hours per day writing with a pen, keyboard, or whiteboard (Table 1).

**Table 1:** Sociodemographic and clinical characteristics of the participants N: frequency, SD: standard deviation, CTS: carpal tunnel syndrome.

Sociodemographic and clinical characteristics		N	(%)
Age (Mean ± SD)		43.3 ± 6.5	
Height (cm) (Mean ± SD)		158.7 ± 18.2	
Weight in (kg) (Mean ± SD)	73.2 ± 15.1	73.2 ± 15.1	
Gender	Male	141	(42.0%)
	Female	195	(58.0%)
If you are female, are you pregnant?	Yes	4	(2.1%)
	No	191	(97.9%)
Marital status	Single	34	(10.1%)
	Married	284	(84.5%)
	Divorced/Widowed	18	(5.4%)
Smoking status	Yes	27	(8.0%)
	No	309	(92.0%)
Do you practice sport?	Yes	169	(50.3%)
	No	167	(49.7%)
Do you use your right or left hand?	Right hand	310	(92.3%)
	Left hand	26	(7.7%)
Are you currently teaching:	In-person	328	(97.6%)
	Remote	8	(2.4%)
What level are you teaching?	Kindergarten	4	(1.2%)
	Primary stage	145	(43.2%)
	High school	113	(33.6%)
	Intermediate stage	74	(22.0%)
Work status	On the job	321	(95.5%)
	Retired	15	(4.5%)
If you are retired, how long have you been retired by years? (Mean ± SD)		3.2 ± 2.3	
How long have you been working for years? (Mean ± SD)		18.1 ± 8.3	
How much time do you spend writing with a pen, keyboard, or whiteboard during the day by hours? (Mean ± SD)		3.2 ± 2.6	
Carpal tunnel syndrome	Yes	27	(8.0%)
	No	309	(92.0%)
If you have been diagnosed before, how long have you been diagnosed? (Mean ± SD)		5.6 ± 4.9	
If you were diagnosed before, what was the treatment?	Surgical	7	(6.4%)
	Non-surgical (conservative treatment)	16	(14.5%)
	No treatment was done	87	(79.1%)
If you have been treated, has your CTS returned or recurred?	No	91	(84.3%)
	Yes	17	(15.7%)
Do you suffer from any of the following diseases?	Diabetes	31	(9.2%)
	Hypertension	43	(12.8%)
	Hypothyroidism	19	(5.7%)
	Rheumatoid arthritis	16	(4.8%)
	Other diseases	17	(5.1%)
	None	210	(62.5%)

The prevalence of CTS among participants was 8.0% (n = 27). Among those diagnosed with CTS, the mean duration since diagnosis was 5.6 ± 4.9 years. Seven (6.4%) had undergone surgical treatment, 16 (14.5%) had received conservative treatment, and 87 (79.1%) had not received any treatment. Of those treated, 17 (15.7%) reported recurrence of CTS symptoms. Comorbidities were also assessed, with 31 (9.2%) participants reporting diabetes, 43 (12.8%) hypertension, 19 (5.7%) hypothyroidism, and 16 (4.8%) rheumatoid arthritis. Other diseases were reported by 17 (5.1%) participants, whereas 210 (62.5%) did not report any comorbidities (Table 1).

Table 2 shows the participants' responses to the BCTQ regarding symptom severity, revealing critical insights into the prevalence and intensity of CTS symptoms among the teachers in Jazan. Most participants (64.9%) reported no hand or wrist pain at night. Regarding sleep disruption due to hand or wrist pain in the past two weeks, 74.1% of participants indicated that they never woke up because of pain. A smaller fraction (14.6%) woke up once, and 7.7% woke up two to three times. Only 1.8% of the participants reported waking up four to five times or more than five times. During the day, 67.3% of the participants did not experience hand or wrist pain. Regarding the frequency of pain during the day, 66.7% of the participants experienced no pain, while 24.4% reported pain once or twice a day. Pain occurring three to five times a day was noted by 4.5%, more than five times by 1.2%, and constant pain by 3.3%.

**Table 2:** Response of the participants on BCTQ (Part 1) regarding Symptom Severity Scale BCTQ: Boston Carpal Tunnel Syndrome Questionnaire.

Symptom severity scale		N	(%)
How severe is the hand or wrist pain during the night?	No pain (1)	218	(64.9%)
	Mild pain (2)	60	(17.9%)
	Moderate pain (3)	39	(11.6%)
	Severe pain (4)	13	(3.9%)
	Very severe pain (5)	6	(1.8%)
How often do you wake up from sleep due to hand or wrist pain during the last two weeks (once a day)?	Never (1)	249	(74.1%)
	Once (2)	49	(14.6%)
	Two to three times (3)	26	(7.7%)
	Four to five times (4)	6	(1.8%)
	More than five times (5)	6	(1.8%)
Do you suffer from hand or wrist pain during the day?	No pain (1)	226	(67.3%)
	Mild pain (2)	69	(20.5%)
	Moderate pain (3)	30	(8.9%)
	Severe pain (4)	8	(2.4%)
	Very severe pain (5)	3	(0.9%)
How often do you experience hand or wrist pain during the day (once a day)?	Never (1)	224	(66.7%)
	Once or twice a day (2)	82	(24.4%)
	Three to five times a day (3)	15	(4.5%)
	More than five times a day (4)	4	(1.2%)
	Constant pain (5)	11	(3.3%)
If you feel pain during the day, approximately how long does the pain last (in minutes)?	I have no pain during the day (1)	229	(68.2%)
	Less than 10 minutes (2)	64	(19.0%)
	From 10 to 60 minutes (3)	23	(6.8%)
	More than 60 minutes (4)	9	(2.7%)
	The pain persists during the day (5)	11	(3.3%)
Do you suffer from numbness (loss of feeling in your hand)?	No numbness (1)	221	(65.8%)
	Mild numbness (2)	78	(23.2%)
	Moderate numbness (3)	24	(7.1%)
	Severe numbness (4)	8	(2.4%)
	Very severe numbness (5)	5	(1.5%)
Do you suffer from hand or wrist weakness?	There is no weakness (1)	241	(71.7%)
	Mild weakness (2)	62	(18.5%)
	Medium weakness (3)	23	(6.8%)
	Severe weakness (4)	8	(2.4%)
	Very severe weakness (5)	2	(0.6%)
Do you suffer from a feeling of tingling in your hand?	No numbness (1)	176	(52.4%)
	Mild numbness (2)	110	(32.7%)
	Moderate numbness (3)	35	(10.4%)
	Severe numbness (4)	10	(3.0%)
	Very severe numbness (5)	5	(1.5%)
How severe is the numbness (loss of sensation) or tingling at night?	No numbness or tingling (1)	202	(60.1%)
	Light (2)	90	(26.8%)
	Medium (3)	30	(8.9%)
	Severe (4)	12	(3.6%)
	Very severe (5)	2	(0.6%)
How often do you wake up at night due to hand numbness or numbness during the last two weeks?	Never (1)	234	(69.6%)
	Once (2)	57	(17.0%)
	Two to three times (3)	34	(10.1%)
	Four to five times (4)	2	(0.6%)
	More than five times (5)	9	(2.7%)
Do you have difficulty holding or using small objects such as a pen or key?	No difficulty (1)	272	(81.0%)
	Mild difficulty (2)	45	(13.4%)
	Medium difficulty (3)	11	(3.3%)
	Extreme difficulty (4)	5	(1.5%)
	Very difficult (5)	3	(0.9%)

When asked about the duration of the daytime pain episodes, 68.2% reported no pain. Pain lasting less than 10 minutes was reported by 19.0%, pain lasting 10 to 60 minutes by 6.8%, pain lasting more than 60 minutes by 2.7%, and persistent pain throughout the day by 3.3%. Regarding numbness, 65.8% of the participants did not experience numbness in their hands. Mild numbness was reported by 23.2%, moderate numbness by 7.1%, severe numbness by 2.4%, and severe numbness by 1.5% of participants. Finally, when asked about difficulty holding or using small objects such as a pen or key, 81.0% of the participants reported no difficulty, 13.4% reported mild difficulty, 3.3% medium difficulty, 1.5% extreme difficulty, and 0.9% very severe difficulty.

Table 3 shows the participants' responses to the BCTQ regarding the functional status scale, revealing that the majority reported minimal difficulty in various tasks. Specifically, for tasks such as writing, 79.8% of participants indicated no difficulty, with only 13.7% reporting mild difficulty, 5.1% moderate difficulty, and 1.2% extreme difficulty, while a mere 0.3% could not perform the activity. Similarly, when it came to buttoning clothes, 81.8% of participants experienced no difficulty. Holding a book while reading posed little challenge, as 79.2% reported no difficulty, 14.6% reported mild difficulty, 5.4% reported moderate difficulty, and 0.9% reported extreme difficulty, with no participants indicating a complete inability to perform the activity. Household chores were manageable for the majority, as 71.7% reported no difficulty, 16.4% reported mild difficulty, 9.2% reported moderate difficulty, 1.8% had extreme difficulty, and 0.9% were unable to perform chores. Toting shopping bags was relatively easy; 68.8% experienced no difficulty, 16.1% experienced mild difficulty, 10.1% experienced moderate difficulty, 4.5% experienced extreme difficulty, and 0.6% were unable to perform the activity.

**Table 3:** Response of the participants on BCTQ (Part 2) regarding Functional Status Scale BCTQ: Boston Carpal Tunnel Syndrome Questionnaire.

Functional status scale	No difficulty		Mild difficulty		Moderate difficulty		Extreme difficulty		Cannot perform the activity at all	
	N	(%)	N	(%)	N	(%)	N	(%)	N	(%)
Writing	268	(79.8%)	46	(13.7%)	17	(5.1%)	4	(1.2%)	1	(0.3%)
Buttoning clothes	275	(81.8%)	43	(12.8%)	12	(3.6%)	6	(1.8%)	0	(0.0%)
Holding the book while reading	266	(79.2%)	49	(14.6%)	18	(5.4%)	3	(0.9%)	0	(0.0%)
Holding the phone	251	(74.7%)	59	(17.6%)	20	(6.0%)	6	(1.8%)	0	(0.0%)
Opening cans	245	(72.9%)	60	(17.9%)	20	(6.0%)	10	(3.0%)	1	(0.3%)
Household chores	241	(71.7%)	55	(16.4%)	31	(9.2%)	6	(1.8%)	3	(0.9%)
Tote shopping bags	231	(68.8%)	54	(16.1%)	34	(10.1%)	15	(4.5%)	2	(0.6%)
Bathing and dressing	273	(81.3%)	39	(11.6%)	15	(4.5%)	9	(2.7%)	0	(0.0%)

Table 4 presents the associations between various sociodemographic, clinical, and lifestyle factors and the SSS and FSS scores among the study participants. The median SSS score was significantly higher among females compared to males (15.0 vs. 12.0, p < 0.001), and a similar trend was observed for the FSS score (8.0 vs. 8.0, p < 0.001). Marital status was significantly associated with FSS score (p = 0.009), with divorced/widowed participants having a higher median score (11.0) than married (8.0) and single (9.0) participants.

**Table 4:** Association of sociodemographic, clinical characteristics, and lifestyle with SSS score and FSS score SSS: Symptom Severity Scale, FSS: Functional Status Scale, CTS: carpal tunnel syndrome. *p value < 0.05.

Sociodemographic, clinical characteristics, and lifestyle		Symptom severity score		Functional status scale score	
		Median (IQR)	p value	Median (IQR)	p value
Gender	Male	12.0 (11.0-17.0)	<0.001*	8.0 (8.0-9.0)	<0.001*
	Female	15.0 (11.0-21.0)		8.0 (8.0-14.0)	
If you are female, are you pregnant?	Yes	15.0 (12.0-33.5)	0.754	8.0 (8.0-16.0)	0.628
	No	15.0 (11.0-21.0)		8.0 (8.0-14.0)	
Marital status	Single	16.5 (11.0-25.0)	0.120	9.0 (8.0-14.0)	0.009*
	Married	13.0 (11.0-19.0)		8.0 (8.0-11.0)	
	Divorced/Widowed	14.0 (12.0-21.0)		11.0 (8.0-18.0)	
Smoking status	Yes	13.0 (11.0-23.0)	0.859	8.0 (8.0-10.0)	0.878
	No	13.0 (11.0-19.0)		8.0 (8.0-11.0)	
Do you practice sport?	Yes	13.0 (11.0-19.0)	0.366	8.0 (8.0-11.0)	0.031*
	No	13.0 (11.0-20.0)		8.0 (8.0-13.0)	
Are you use right or left hand?	Right hand	13.0 (11.0-20.0)	0.236	8.0 (8.0-11.0)	0.633
	Left hand	13.5 (12.0-19.0)		8.0 (8.0-14.0)	
Are you currently teaching:	In person	13.0 (11.0-20.0)	0.298	8.0 (8.0-11.0)	0.658
	Remote	17.5 (12.0-23.5)		8.5 (8.0-13.0)	
What level are you teaching?	Kindergarten	17.5 (11.5-25.5)	0.241	8.5 (8.0-12.5)	0.286
	Primary stage	13.0 (11.0-19.0)		8.0 (8.0-12.0)	
	High school	13.0 (11.0-17.0)		8.0 (8.0-10.0)	
	Intermediate stage	14.5 (11.0-22.0)		8.0 (8.0-15.0)	
Work status	On the job	13.0 (11.0-20.0)	0.649	8.0 (8.0-11.0)	0.338
	Retired	12.0 (11.0-18.0)		8.0 (8.0-9.0)	
CTS	Yes	30.0 (22.0-43.0)	<0.001*	19.0 (15.0-24.0)	<0.001*
	No	13.0 (11.0-18.0)		8.0 (8.0-10.0)	
If you were diagnosed before, what was the treatment?	Surgical	32.0 (31.0-47.0)	<0.001*	24.0 (16.0-25.0)	<0.001*
	Non-surgical (conservative treatment)	24.5 (19.0-40.0)		17.5 (9.0-27.0)	
	No treatment was done	13.0 (11.0-21.0)		8.0 (8.0-13.0)	
If you have been treated, has your CST returned or recurred?	No	13.0 (11.0-19.0)	<0.001*	8.0 (8.0-13.0)	<0.001*
	Yes	28.0 (24.0-45.0)		22.0 (12.0-27.0)	
Do you suffer from any of the following diseases?	Diabetes	16.0 (11.0-23.0)	0.002*	8.0 (8.0-16.0)	0.005*
	Hypertension	12.0 (11.0-18.0)		8.0 (8.0-12.0)	
	Hypothyroidism	15.0 (11.0-23.0)		10.0 (8.0-19.0)	
	Rheumatoid arthritis	22.0 (13.5-25.0)		9.5 (8.0-17.5)	
	Other	21.0 (12.0-24.0)		9.0 (8.0-15.0)	
	None	13.0 (11.0-17.0)		8.0 (8.0-10.0)	
Which hand are you suffering from?	Right	12.0 (11.0-18.0)	0.013*	8.0 (8.0-11.0)	0.763
	Left	14.0 (11.0-19.0)		8.0 (8.0-11.0)	
	Both	15.0 (11.0-22.0)		8.0 (8.0-14.0)	
If you have any of these symptoms, which doctor do you see?	Family doctor	13.0 (11.0-18.0)	0.361	8.0 (8.0-10.0)	0.233
	Orthopedic surgeon	14.0 (11.0-21.0)		8.0 (8.0-15.0)	
	Internal medicine doctor	14.0 (11.0-22.0)		8.0 (8.0-16.0)	
	Plastic surgeon	11.0 (11.0-46.0)		8.0 (8.0-25.0)	

Engaging in regular physical activity was associated with a lower median FSS score (8.0 vs. 8.0, p = 0.031). Participants diagnosed with CTS had significantly higher median SSS and FSS scores than those without CTS (30.0 vs. 13.0 and 19.0 vs. 8.0, respectively; both p < 0.001). Among participants diagnosed with CTS, the type of treatment received was significantly associated with both SSS and FSS scores (p < 0.001). Participants who underwent surgical treatment had the highest median scores (SSS: 32.0, FSS: 24.0), followed by those who received conservative treatment (SSS: 24.5, FSS: 17.5), and those who did not receive any treatment (SSS, 13.0; FSS, 8.0). Participants who experienced a recurrence of CTS symptoms after treatment had significantly higher median SSS and FSS scores than those who did not (28.0 vs. 13.0 and 22.0 vs. 8.0, respectively; both p < 0.001).

The presence of comorbidities was significantly associated with both SSS and FSS scores (p = 0.002 and p = 0.005, respectively). Participants with rheumatoid arthritis had the highest median SSS score (22.0), whereas those with hypothyroidism had the highest median FSS score (10.0). The hand affected by CTS symptoms was significantly associated with the SSS (p = 0.013), with participants experiencing symptoms in both hands having the highest median score (15.0).

These findings highlight the importance of considering various sociodemographic, clinical, and lifestyle factors when assessing CTS severity and functional impact among teachers. The significant associations observed between these factors and SSS and FSS scores underscore the need for targeted interventions and management strategies to address the specific needs of different subgroups within this population.

Table 5 presents the correlations between various continuous sociodemographic variables and the SSS and FSS scores among the study participants. A significant negative correlation was observed between participant height and both the SSS (r = -0.174, 95% CI: -0.280 to -0.064, p = 0.002) and FSS scores (r = -0.141, 95% CI: -.248 to -0.030, p = 0.011). This suggests that taller individuals may experience less severe symptoms and a better functional status related to CTS. The amount of time spent writing with a pen, keyboard, or whiteboard during the day was positively correlated with both the SSS (r = 0.237, 95% CI: 0.126 to 0.342, p < 0.001) and FSS scores (r = 0.217, 95% CI: 0.106 to 0.324, p < 0.001). This finding indicates that increased time spent on writing activities may be associated with more severe symptoms and greater functional impairment among CTS teachers.

**Table 5:** Correlation of continuous variables with SSS score and FSS score SSS: Symptom Severity Scale, FSS: Functional Status Scale. *p < 0.05 considered significant.

Sociodemographic	Symptom severity scale score		Functional status scale score	
	Correlation coefficient (95% CI)	p-value	Correlation coefficient (95% CI)	p-value
Age	-0.070 (-0.179 to 0.041)	0.203	-0.003 (-0.113 to 0.108)	0.958
Length	-0.174 (-0.280 to -0.064)	0.002*	-0.141 (-0.248 to -0.030)	0.011*
Weight	0.022 (-0.088 to 0.132)	0.687	0.064 (-0.047 to 0.173)	0.245
If you are retired, how long have you been retired?	0.304 (-0.241 to 0.703)	0.252	-0.171 (-0.625 to 0.369)	0.526
How long have you been working?	-0.016 (-0.129 to 0.097)	0.769	0.081 (-0.032 to 0.192)	0.150
How much time do you spend writing with a pen, keyboard, or whiteboard during the day?	0.237 (0.126 to 0.342)	<0.001*	0.217 (0.106 to 0.324)	<0.001*
If you have been diagnosed before, how long have you been diagnosed?	0.293 (-0.127 to 0.625)	0.155	0.168 (-0.255 to 0.537)	0.422

No significant correlations were found between age, weight, retirement duration, work duration, or time since CTS diagnosis and either the SSS or FSS scores (all p > 0.05). These results suggest that these factors may not have a strong linear relationship with symptom severity or functional status in this population. The significant correlations observed between height and writing time and SSS and FSS scores provide valuable insights into potential risk factors for CTS severity and functional impairment among teachers. These findings highlight the importance of considering ergonomic factors and modifying work practices to reduce the impact of CTS in this population.

Table 6 presents the associations between various factors and CTS diagnoses among the study participants. The amount of time spent writing with a pen, keyboard, or whiteboard during the day was significantly higher among those diagnosed with CTS compared to those without CTS (4.8 ± 2.8 hours vs. 3.1 ± 2.5 hours, p = 0.003). This finding suggests that increased exposure to writing activities may be a risk factor for CTS. Among participants previously diagnosed with CTS, the type of treatment received was significantly associated with current CTS diagnosis (p < 0.001). A higher proportion of participants who underwent surgical (71.4%) or conservative treatment (75.0%) were currently diagnosed with CTS than those who did not receive any treatment (11.5%). Additionally, a significantly higher proportion of participants who experienced recurrence of CTS symptoms after treatment were currently diagnosed with CTS compared to those who did not experience recurrence (76.5% vs. 11.0%, p < 0.001). These results highlight the importance of effective treatment and post-treatment management to prevent CTS recurrence.

**Table 6:** Association of participants already diagnosed with carpel tunnel syndrome (CTS) with various factors N: frequency, SD: standard deviation. *p < 0.05 considered significant.

Factors		Diagnosed with CTS				
		Yes		No		p-value
		N	(%)	N	(%)	
Gender	Male	8	(5.7%)	133	(94.3%)	0.176
	Female	19	(9.7%)	176	(90.3%)	
If you are female, are you pregnant?	Yes	1	(25.0%)	3	(75.0%)	0.339
	No	18	(9.4%)	173	(90.6%)	
Age (Median (IQR))		44.0 (40.0-47.0)		44.0 (40.0-48.0)		0.851
Length in (cm) (Mean ± SD)		157.3 ± 10.4		158.8 ± 18.7		0.666
Weight in (kg) (Mean ± SD)		74.5 ± 14.8		73.1 ± 15.1		0.637
Marital status	Single	4	(11.8%)	30	(88.2%)	0.424
	Married	21	(7.4%)	263	(92.6%)	
	Divorced/Widowed	2	(11.1%)	16	(88.9%)	
Smoking status	Yes	4	(14.8%)	23	(85.2%)	0.255
	No	23	(7.4%)	286	(92.6%)	
Do you practice sport?	Yes	14	(8.3%)	155	(91.7%)	0.866
	No	13	(7.8%)	154	(92.2%)	
Are you use right or left hand?	Right hand	25	(8.1%)	285	(91.9%)	1.000
	Left hand	2	(7.7%)	24	(92.3%)	
Are you currently teaching:	In-person	25	(7.6%)	303	(92.4%)	0.129
	Remote	2	(25.0%)	6	(75.0%)	
What level are you teaching?	Kindergarten	0	(0.0%)	4	(100.0%)	0.727
	Primary stage	14	(9.7%)	131	(90.3%)	
	High school	7	(6.2%)	106	(93.8%)	
	Intermediate stage	6	(8.1%)	68	(91.9%)	
Work status	On the job	25	(7.8%)	296	(92.2%)	0.343
	Retired	2	(13.3%)	13	(86.7%)	
If you are retired, how long have you been retired? (Median (IQR))		4.5 (4.0-5.0)		2.0 (1.0-4.0)		0.267
How long have you been working? (Mean ± SD)		19.5 ± 8.9		18.0 ± 8.3		0.355
How much time do you spend writing with a pen, keyboard, or whiteboard during the day? (Mean ± SD)		4.8 ± 2.8		3.1 ± 2.5		0.003*
If you were diagnosed before, what was the treatment?	Surgical	5	(71.4%)	2	(28.6%)	<0.001*
	Non-surgical (conservative treatment)	12	(75.0%)	4	(25.0%)	
	No treatment was done	10	(11.5%)	77	(88.5%)	
If you have been treated, has your CTS returned or recurred?	No	10	(11.0%)	81	(89.0%)	<0.001*
	Yes	13	(76.5%)	4	(23.5%)	
Do you suffer from any of the following diseases?	Diabetes	6	(19.4%)	25	(80.6%)	0.026*
	Hypertension	2	(4.7%)	41	(95.3%)	
	Hypothyroidism	4	(21.1%)	15	(78.9%)	
	Rheumatoid arthritis	1	(6.3%)	15	(93.8%)	
	other	2	(11.8%)	15	(88.2%)	
	None	12	(5.7%)	198	(94.3%)	
Which hand are you suffering from?	Right	16	(7.9%)	186	(92.1%)	0.850
	Left	5	(6.9%)	67	(93.1%)	
	Both of them	6	(9.7%)	56	(90.3%)	
If you have any of these symptoms, which doctor do you see?	Family doctor	9	(4.7%)	181	(95.3%)	0.014*
	Orthopedic surgeon	16	(13.2%)	105	(86.8%)	
	Internal medicine doctor	1	(4.5%)	21	(95.5%)	
	Plastic surgeon	1	(33.3%)	2	(66.7%)	
If you have been diagnosed before, how long have you been diagnosed? (Mean ± SD)		6.1 ± 5.3		3.8 ± 1.8		0.358

The presence of comorbidities was significantly associated with CTS diagnosis (p = 0.026). Participants with hypothyroidism (21.1%) and diabetes (19.4%) had the highest proportion of CTS diagnoses, compared to those with other comorbidities or no comorbidities. This finding underscores the need to consider the management of underlying health conditions for the prevention and treatment of CTS. The type of healthcare provider consulted for CTS symptoms was also significantly associated with CTS diagnosis (P = 0.014). Participants who consulted an orthopedic surgeon had the highest proportion of CTS diagnoses (13.2%), followed by those who consulted a plastic surgeon (33.3%), although the latter group had a small sample size. This suggests that seeking care from specialists may be associated with a higher likelihood of CTS diagnosis, possibly because of their expertise in diagnosing and managing the condition.

No significant associations were found between CTS diagnosis and factors such as gender, pregnancy status, age, height, weight, marital status, smoking status, physical activity, hand dominance, work location, teaching mode, teaching level, work status, retirement duration, work duration, or affected hand (all p > 0.05). These findings provide valuable insights into the factors associated with CTS diagnoses among teachers. The significant associations observed between writing time, treatment history, comorbidities, and healthcare provider type highlight the potential areas for intervention and management strategies. However, the lack of significant associations with other factors suggests that the development and diagnosis of CTS may involve complex interactions between various risk factors and individual characteristics.

Table 7 shows the binary logistic regression analysis aimed at predicting known CTS diagnoses, and several continuous variables were assessed for predictive significance. The results indicated that age, length, weight, retirement duration, and work duration did not significantly predict the likelihood of a known diagnosis of CTS. However, the amount of time spent writing with a pen, keyboard, or whiteboard during the day emerged as a significant predictor (p = 0.018, OR = 1.151, 95% CI: 1.024-1.295). This suggests that for every unit increase in the time spent writing, there was an approximately 15.1% increase in the odds of having a CTS diagnosis.

**Table 7:** Binary logistic regression model with continuous variables as predictors of known diagnoses of carpel tunnel syndrome CI: confidence interval. *p < 0.05 considered significant.

Predictors	p-value	Odds ratio	95% CI
Age	0.940	1.002	0.943-1.066
Length (cm)	0.666	0.996	0.977-1.015
Weight (kg)	0.636	1.006	0.981-1.032
If you are retired, how long have you been retired?	0.382	1.322	0.706-2.476
How long have you been working?	0.354	1.024	0.974-1.076
How much time do you spend writing with a pen, keyboard, or whiteboard during the day?	0.018*	1.151	1.024-1.295

## Discussion

Compression of the median nerve in the carpal tunnel causes CTS, a common hand and wrist musculoskeletal ailment [3]. Teachers are at an increased risk of CTS because their jobs entail a lot of writing, typing, and computer use [1]. The current study found that 8.0% of teachers had CTS symptoms, similar to a study in Riyadh, Saudi Arabia, which found 9.1% [6]. This prevalence is greater than that reported in the general population, which ranges between 1% and 5% [12], indicating an elevated risk of CTS among instructors.

Several characteristics have been identified as being substantially linked to increased CTS symptom severity and functional impairment. Females had significantly higher median SSS and FSS scores than men (15.0 vs. 12.0, p < 0.001; 8.0 vs. 8.0, p < 0.001, respectively). This conclusion is consistent with prior research that has found a greater frequency of CTS in females [13,14]. The greater occurrence in females may be due to hormonal events, such as pregnancy and menopause, which can cause fluid retention and increased pressure in the carpal tunnel [15]. Furthermore, females often have lower carpal tunnel dimensions than males, which may enhance their vulnerability to CTS [16]. Furthermore, females are more likely to report symptoms and seek medical assistance for CTS than males [17].

This research implies that geographical characteristics, such as work environment and occupational pressures, may impact the onset of CTS symptoms in teachers. Jazan is an area in southwest Saudi Arabia that is noted for its hot and humid environment [18]. High temperatures and humidity may exacerbate the development of CTS symptoms by increasing fluid retention and edema in the wrist [19]. Furthermore, the educational system in Jazan may have unique needs or practices that enhance the risk of CTS among instructors, such as longer working hours, heavier workload, or fewer ergonomic resources [1]. More studies are needed to understand the variables influencing this connection in the Jazan region and to create focused strategies to reduce the incidence of CTS among teachers in this area.

Increased writing time was strongly associated with the SSS (r = 0.237, p < 0.001) and FSS scores (r = 0.217, p < 0.001). This conclusion is consistent with earlier research that has highlighted repeated hand motions and extended writing as risk factors for CTS development [20,21]. Writing requires repeated wrist flexion and extension, which can increase pressure on the median nerve and result in the development of CTS symptoms [22]. Furthermore, extended writing sessions without sufficient pauses or suitable ergonomic placement may increase the risk of CTS [23]. Teachers frequently spend a substantial amount of their workdays writing on the chalkboard, grading papers, and planning lessons, which may contribute to the higher occurrence of CTS symptoms in this population [24]. Interventions targeted at lowering writing time, supporting correct ergonomic techniques, and encouraging frequent breaks may help reduce the risk of CTS in teachers [1].

Comorbidities, such as diabetes and hypothyroidism, were associated with considerably higher SSS and FSS scores. Those with diabetes had a median SSS of 16.0 (p = 0.002) and a median FSS of 8.0 (p = 0.005), whereas those with hypothyroidism had a median SSS of 15.0 (p = 0.002) and a median FSS of 10.0 (p = 0.005). These findings are like those of prior research that identified diabetes and hypothyroidism as risk factors for CTS [25]. Diabetes can produce CTS through a variety of pathways, including the development of advanced glycation end-products, increased oxidative stress, and microvascular damage [26]. Hypothyroidism, on the other hand, might exacerbate CTS by inducing fluid retention and swelling in the carpal tunnel, putting more pressure on the median nerve [27]. Furthermore, diabetes and hypothyroidism can cause peripheral neuropathy, exacerbating the symptoms [28]. Regular screening and treatment of these comorbidities may aid in lowering the risk and severity of CTS in teachers [29].

Height was negatively correlated with symptom severity (r = -0.174, p = 0.002) and functional impairment (r = -0.141, p = 0.011). This research implies that taller people may have less severe CTS symptoms and higher functional status. Previous research has found similar connections, with shorter height being a risk factor for CTS [19,30]. The specific processes behind this connection are unknown; however, they may be linked to variations in carpal tunnel diameter and biomechanical characteristics [22]. Taller individuals may have greater carpal tunnel dimensions, lowering the incidence of median nerve compression [31]. Furthermore, taller people may have longer arms and forearms, which may affect the biomechanics of the wrist and hand during repeated tasks such as writing [32]. However, it is crucial to remember that height is a non-modifiable risk factor, and treatments should focus on modifiable variables, such as ergonomics and work habits, to minimize the risk of CTS in teachers of all heights [33].

Participants with CTS who underwent surgical therapy had the greatest median SSS (32.0) and FSS (24.0), followed by those who received conservative treatment (SSS: 24.5, FSS: 17.5) and those who did not receive any treatment (SSS: 13.0, FSS: 8.0) (p < 0.001). This discovery may appear contradictory, given that surgical therapy is expected to yield better results than conservative treatment or no treatment. However, it is crucial to note that individuals who underwent surgical therapy reported more severe CTS symptoms and functional impairment at the start, which may have led them to seek surgical intervention [34]. Furthermore, the cross-sectional design of this study did not allow the evaluation of long-term outcomes after surgical therapy. Previous research has shown that surgical therapy can significantly improve CTS symptoms and functional status, especially in cases of severe CTS [35]. However, the outcome of surgical therapy depends on several factors, including the timing of the operation, surgical method employed, and postoperative rehabilitation [36].

Participants with symptom recurrence following therapy had substantially higher median SSS (28.0) and FSS (22.0) ratings than those without recurrence (13.0 and 8.0, respectively) (p < 0.001). This study emphasizes the need for appropriate post-treatment care and follow-up to prevent CTS recurrence and to sustain long-term functional outcomes. Previous research has reported recurrence rates ranging from 4% to 57% after surgical therapy for CTS [37]. Incomplete transverse carpal ligament release, scar tissue development, and underlying medical problems such as diabetes and rheumatoid arthritis have all been linked to an increased risk of recurrence [38]. Furthermore, insufficient postoperative rehabilitation and return to repetitive hand tasks too soon after surgery may lead to the recurrence of CTS symptoms [39]. Comprehensive post-treatment management techniques, including patient education, ergonomic changes, and phased return to work, may help minimize the chance of recurrence and improve long-term results [40].

A binary logistic regression analysis found that increased writing time was a significant predictor of CTS diagnosis (OR = 1.151, 95% CI: 1.024-1.295, p = 0.018). This study adds to the evidence that repeated hand motions and extended writing contribute to the development of CTS in teachers. Previous research has found similar relationships, underscoring the need for ergonomic treatments and work practice improvements to lower the incidence of CTS in this occupational group [2]. Ergonomic therapies, such as using ergonomic pens, placing the wrist and hand correctly when writing, and using assistive equipment such as wrist supports, may help minimize the incidence of CTS among instructors. Furthermore, taking regular breaks, stretching exercises, and employing work rotation tactics may help reduce the cumulative pressure on the wrist and hand during continuous writing [41].

The use of a validated questionnaire (BCTQ) to quantify CTS symptoms and functional status, the large sample size, and the examination of sociodemographic, occupational, and clinical features are the strengths of this study. The sample size and inclusion of instructors from diverse educational levels and conditions made the results more applicable to Jazan's teachers. A thorough evaluation of several risk variables illuminates the intricate interaction between individual, occupational, and clinical aspects in teacher CTS development and progression. However, this study's limitations must be considered when assessing the results. The risk factors and CTS symptoms cannot be causally linked in cross-sectional studies. This study showed CTS risk variables; however, long-term studies are needed to prove their temporal association. Self-reported statistics may underestimate the CTS frequency and generate memory bias. Mild or intermittent symptoms may not have been reported, thus underestimating the prevalence of CTS in this population. Objective CTS tests, such as nerve conduction investigations or ultrasound imaging, may have improved the prevalence and severity estimates. The sample was recruited via social media surveys, which may have caused a selection bias. This recruitment technique may have overrepresented younger, tech-savvy instructors who may have distinct risk profiles. Teachers with severe CTS symptoms were more likely to participate; hence, the study overstated the prevalence and severity of CTS. To reduce bias, future research should employ representative samples and objective CTS measurements.

## Conclusions

This study found a high occurrence of CTS symptoms among schoolteachers in Jazan, Saudi Arabia. This study found that there are several sociodemographic, occupational, and clinical characteristics that have a substantial impact on the severity of CTS symptoms and the amount of functional impairment that instructors with CTS suffer. The characteristics that contribute to this include being female, spending more time writing, having comorbidities such as diabetes and hypothyroidism, and being tall. Individuals who were diagnosed with CTS showed a correlation between surgical therapy and the return of symptoms following treatment, which was linked to greater severity and impairment ratings.

The study results emphasize the necessity for focused treatments and management techniques to prevent and alleviate the consequences of CTS among instructors. These interventions may encompass ergonomic adjustments, prompt identification and proper treatment of CTS, and the management of underlying medical disorders. The strong correlation between extended writing duration and CTS diagnosis underscores the necessity of addressing ergonomic variables and adjusting work methods to mitigate the likelihood of CTS among educators.

Additional investigation is required to examine the precise processes that underlie the connections revealed as well as to create and assess the efficacy of focused treatments for the prevention and treatment of CTS in this occupational cohort. By prioritizing the prevention and management of CTS among teachers, we can guarantee that this crucial workforce remains in good health, maintains high productivity, and continues to fulfill their key role in educating future generations.
